# Transactions of the Medical and Chirurgical Faculty of the State of Maryland

**Published:** 1889-02

**Authors:** 


TRANSACTIONS OF THE MEDICAL AND CHIRURGICAL FACULTY OF THE STATE OF MARYLAND. Nineteenth Annual Session, held at Baltimore, April, 1888.

The Maryland Faculty is one of the most substantial medical organizations in this country, if one is to jndge trom its volume of Transactions. John Morris, M. D., is President, and G. Lane Taneyhill, M. D., Recording Secretary. The minutes show a working society. The papers are good but some of them are rather long for a busy doctor to wade through.—B.

